# Identification of a potential novel biomarker in intervertebral disk degeneration by bioinformatics analysis and experimental validation

**DOI:** 10.3389/fimmu.2023.1136727

**Published:** 2023-05-30

**Authors:** Zhao Yang, Zhen-Zhen Yuan, Xin-Long Ma

**Affiliations:** Department of Orthopedics, Tianjin Hospital, Tianjin, China

**Keywords:** intervertebral disc degeneration, biomarker, nucleus pulposus cell, immune cell infiltration, bioinformatics, machine learning algorithms

## Abstract

**Background:**

Intervertebral disk degeneration (IVDD) is a major cause of low back pain and one of the most common health problems all over the world. However, the early diagnosis of IVDD is still restricted. The purpose of this study is to identify and validate the key characteristic gene of IVDD and analyze its correlation with immune cell infiltration.

**Methods:**

3 IVDD-related gene expression profiles were downloaded from the Gene Expression Omnibus database to screen for differentially expressed genes (DEGs). Gene Ontology (GO) and gene set enrichment analysis (GSEA) were conducted to explore the biological functions. Two machine learning algorithms were used to identify characteristic genes, which were tested to further find the key characteristic gene. The receiver operating characteristic curve was performed to estimate the clinical diagnostic value of the key characteristic gene. The excised human intervertebral disks were obtained, and the normal nucleus pulposus (NP) and degenerative NP were carefully separated and cultured *in vitro*. The expression of the key characteristic gene was validated by real-time quantitative PCR (qRT-PCR). The related protein expression in NP cells was detected by Western blot. Finally, the correlation was investigated between the key characteristic gene and immune cell infiltration.

**Results:**

A total of 5 DEGs, including 3 upregulated genes and 2 downregulated genes, were screened between IVDD and control samples. GO enrichment analysis showed that DEGs were enriched to 4 items in BP, 6 items in CC, and 13 items in MF. They mainly included the regulation of ion transmembrane transport, transporter complex, and channel activity. GSEA suggested that the cell cycle, DNA replication, graft versus host disease, and nucleotide excision repair were enriched in control samples, while complement and coagulation cascades, Fc γ R–mediated phagocytosis, neuroactive ligand–receptor interaction, the NOD-like receptor signaling pathway, gap junctions, etc., were enriched in IVDD samples. Furthermore, ZNF542P was identified and tested as key characteristic gene in IVDD samples through machine learning algorithms and showed a good diagnostic value. The results of qRT-PCR showed that compared with normal NP cells, the expression of ZNF542P gene was decreased in degenerated NP cells. The results of Western blot suggested that compared with normal NP cells, the expression of NLRP3 and pro Caspase-1 was increased in degenerated NP cells. Finally, we found that the expression of ZNF542P was positively related to the proportions of T cells gamma delta (γδT cells).

**Conclusion:**

ZNF542P is a potential biomarker in the early diagnosis of IVDD and may be associated with the NOD-like receptor signaling pathway and the infiltration of γδT cells.

## 1 Introduction

Clinically, low back pain is one of the most medical problems in spine surgery affecting 70%–85% of the population worldwide (1). Many causes could lead to low back pain, while intervertebral disk degeneration (IVDD) is considered as the most common reason (2). Currently, the main clinical therapeutic approaches regarding IVDD include conservative treatment and surgery. Symptomatic recurrence often occurs after conservative treatment, while surgery would destabilize the spine. Thus, IVDD has tremendous potential for improvement in early diagnostic aspects (3).

Bioinformatics is a discipline that studies the complex relationship of a large number of biological data, which is characterized by multidisciplinary interdisciplinary, using the internet as the medium and the database as the carrier. It takes advantage of various mathematical knowledge to establish different models and then uses the computer as a tool to store, retrieve, process, and analyze a large amount of biological data obtained from experiments and explains the results with biological knowledge (4). Machine learning is a multifield interdisciplinary subject that has risen in the past 20 years, involving the probability theory, statistics, approximation theory, convex analysis, algorithm complexity theory, and other disciplines. The machine learning algorithm is a kind of method that automatically analyzes and obtains rules from data and uses the rules to predict unknown data (5).

The purpose of our study is to use the RNA expression profiles obtained from the Gene Expression Omnibus (GEO) database to identify the key characteristic gene, validate the gene *via* an *in vitro* experiment, and analyze its correlation with immune cell infiltration in IVDD. In this study, we systematically analyzed gene expression profiles based on bioinformatics and machine learning algorithms between IVDD and healthy patients. In addition, we validated a key characteristic gene as an early diagnostic biomarker in IVDD.

## 2 Materials and methods

### 2.1 Dataset acquisition

3 IVDD-related gene expression profiles were downloaded from the GEO database, (https://www.ncbi.nlm.nih.gov/geo/). The GSE124272 (GPL21185) dataset included 8 patients with lumbar disk prolapse and 8 healthy volunteers. The GSE153761 (GPL22120) dataset included 3 patients with cervical spondylosis myelopathy and 3 healthy subjects. The GSE150408 (GPL21185) dataset including 17 patients with sciatica and 17 healthy volunteers was selected as a validation set. The GSE124272 and GSE153761 datasets were selected as a training set and to screen for differentially expressed genes (DEGs) between IVDD and control samples shown in **Table 1**.

**Table 1:** Sample information for each dataset.

Dataset	Platform	IVDD	Control	Group
GSE124272	GPL21185 Agilent-072363 SurePrint G3 Human GE v3 8x60K Microarray 039494	8	8	Training set
GSE153761	GPL22120 Agilent-078298 human ceRNA array V1.0 4X180K	3	3	Training set
GSE150408	GPL21185 Agilent-072363 SurePrint G3 Human GE v3 8x60K Microarray 039494	17	17	Validation set

### 2.2 Gene expression profile preprocessing

In this study, we downloaded 2 types of file for analysis: “platform” data and “series_matrix_file” data, among which “platform” data included GPL21185 and GPL22120, while “series_matrix_file” data contained GSE124272, GSE153761, and GSE150408. Platform data were used to probe the”series_matrix _file” data changing probe sets into gene symbols. Probe sets without corresponding gene symbols and repeated gene symbols were removed, and the median expression value was retained when multiple probe sets were mapped to the same gene.

### 2.3 Screening for differentially expressed genes

After the expression matrix of GSE124272 and GSE153761 datasets were merged, the data were further corrected and normalized. Genes between IVDD and control samples were screened by R software with the threshold of the adjusted p-value < 0.05 and ∣log2 fold change (FC)∣>0.5 were regarded as DEGs. Moreover, the heat map and volcano plot were plotted to visualize the date.

### 2.4 Gene Ontology and gene set enrichment analysis

Gene Ontology (GO) enrichment analysis was significantly related to the disease and could classify GO terms in 3 categories: biological processes (BP), cellular component (CC), and molecular function (MF). For gene set enrichment analysis (GSEA), it was performed to get more important biological functions or signaling pathways based on all genes and phenotype, which may be neglected by differential analysis. For both analyses, a p-value < 0.05 was considered as statistical significance.

### 2.5 Machine learning algorithms to identify characteristic genes

To further screen characteristic genes related to IVDD, least absolute shrinkage and selection operator (LASSO) logistic regression and support vector machine–recursive feature elimination (SVM-RFE) algorithms, two machine learning approaches, were combined to perform feature selection calculating the point with the smallest error in the training set (GSE124272 and GSE153761) by cross-validation. Next, the overlapping genes in the LASSO logistic regression and the SVM-RFE algorithm were regarded as characteristic genes for further analysis. Furthermore, to screen the key characteristic gene, we further tested the expression levels of characteristic genes in the validation set (GSE150408).

### 2.6 Estimation of clinical diagnostic value of key characteristic genes

To verify whether key characteristic genes could differentiate IVDD samples from control samples in the training set and validation set, the receiver operating characteristic (ROC) curves were plotted to estimate the clinical diagnostic value of key characteristic genes by calculating the area under the ROC curves (AUC).

### 2.7 Isolation and culture of human nucleus pulposus cells

The experimental protocol received approval from the Tianjin Hospital Ethics Committee. All patients participating in this study signed written informed consent allowing the researchers to use intervertebral disk tissues obtained from surgery. The intervertebral disk tissue was obtained from two patients (1 spinal trauma patient and 1 IVDD patient).

The NP tissue was cut into approximately 1 mm^3^ fragment, subsequently digested by 0.25% trypsin and 0.2% type II collagenase at 37°C, and filtered through a 100 μm nylon mesh. After isolation, NP cells were cultured in Dulbecco's Modified Eagle Medium (DMEM)/F12 medium containing 10% fetal bovine serum and penicillin at 37°C in a 5% CO_2_ incubator. The passage 3 (P3) cells were adopted for the following experiments.

### 2.8 Quantitative real-time polymerase chain reaction analysis

Total mRNA from NP cells was extracted with the TRIzol reagent (Invitrogen, USA), and cDNA was synthesized by the Rever Tra Ace qPCR RT Kit (TOYOBO, Japan). Real-time quantitative PCR (qRT-PCR) was performed with SYBR^®^ Green Real-Time PCR Master Mix (TOYOBO, Japan). The GAPDH was used as a housekeeper gene. The ZNF542P and GAPDH PCR primer sequences were shown as follows: ZNF542P Forward (5’-3’) CTGCGAGTCCTCGCCTACT, Backward (5’-3’) CTGCCCAAGAAAGGAGAAACG. GAPDH Forward (5’-3’) GGTGGTCTCCTCTGACTTCAACAG, Backward (5’-3’) GTTGCTGTAGCCAAATTCGTTGTC.

### 2.9 Western blot

Total protein in NP cells was extracted with Radio Immunoprecipitation Assay (RIPA) lysis buffer, and the protein concentration was measured using the BCA Quantitation Kit (Boster, China). Proteins were separated by SDS-PAGE and transferred to polyvinylidene fluoride (PVDF) membranes. The membranes were blocked with 5% skim milk and then incubated with primary antibodies against NLRP3 (1:1,000; Abcam, UK), pro Caspase-1 (1:1,000; Abcam, UK), respectively, overnight at 4°C. The membranes were incubated with horseradish peroxidase–conjugated (HRP) secondary antibodies (1:1,000) for 1 h. Blots were developed using enhanced chemiluminescence (Santa Cruz Biotechnology, USA).

### 2.10 Correlation between key characteristic gene and immune cell infiltration

To further explore the correlation between the key characteristic gene and immune cell infiltration, we assessed the proportion and composition of infiltrating immune cells in IVDD and control samples in the training set by CIBERSORT analysis, a deconvolution algorithm based on a validated leukocyte signature matrix including 547 genes and 22 human immune cell subtypes (6). The difference between IVDD and control samples was shown using a boxplot and a violin plot. The correlation between the key characteristic gene and immune cell infiltration was shown using a scatter plot and a lollipop plot.

### 2.11 Statistical analysis

The statistical analysis and visualization of all data were processed using R language (version 4.1.2, based on corresponding R package). p-value < 0.05 was considered as statistically significant difference. Gene expression data were represented as mean ± standard deviation (±s). Statistical analysis was processed by the *t*-test using software SPSS 21.0.

## 3 Results

### 3.1 Data preprocessing and screening for differentially expressed genes

**Figure 1:**
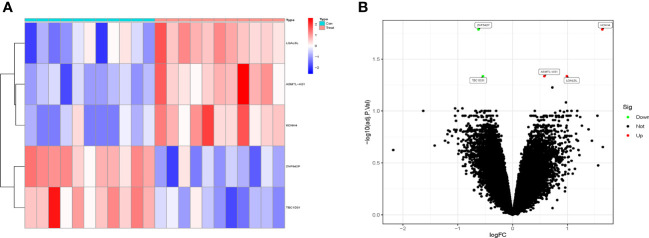
The differentially expressed genes (DEGs) analysis of the training set. **(A)** The heat map of DEGs expression with high expression in red and low expression in blue. **(B)** The volcano plot of DEGs expression showed upregulated genes in red and downregulated genes in green.

The GSE124272 dataset contains 16 samples (25,964 genes), and the GSE153761 dataset contains 6 samples (38,156 genes). After merging the data, the training set contains 22 samples (25,964 genes). After the standardization of the data, the training set contains 22 samples (22,833 genes). The validation set contains 34 samples (25,964 genes). A total of 5 DEGs were screened, including 3 upregulated genes (KCNH4, LGALSL, and ASMTL-AS1) and 2 downregulated genes (ZNF542P and TBC1D31). The heat map and volcano plot of DEGs expression were shown in **Figures 1A, B**.

### 3.2 Gene Ontology and gene set enrichment analyses

**Figure 2:**
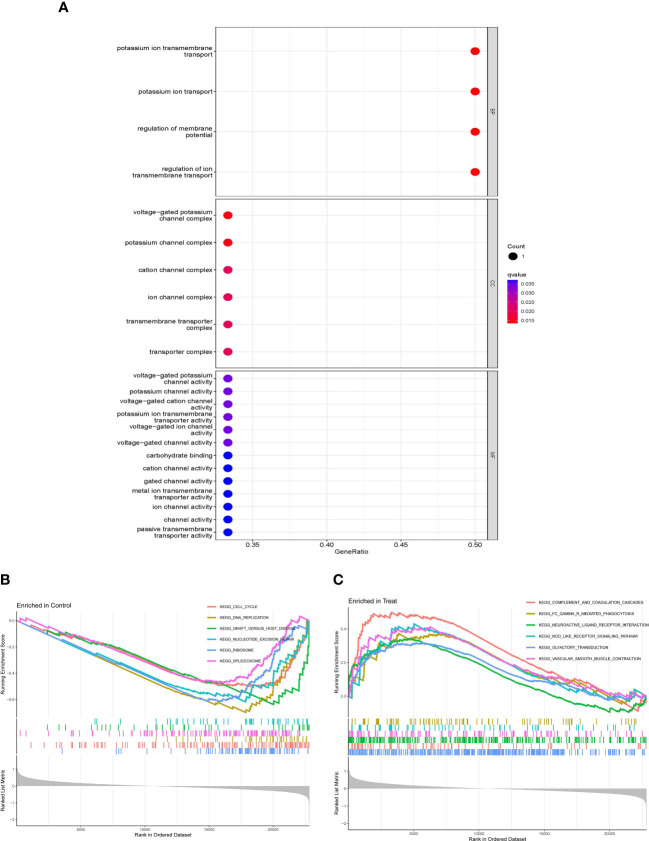
Gene Ontology (GO) enrichment analysis of DEGs and the gene set enrichment analysis (GSEA) of whole gene expressions. **(A)** Bubble plot of GO enrichment analysis, including BP, CC, and MF. **(B, C)** Line graph of GSEA in control and intervertebral disk degeneration (IVDD) samples.

GO enrichment analysis includes 3 diverse categories: BP, CC, and MF. The top terms were selected according to the q-value from small to large, which could be observed in **Figure 2A**. With a total of 4 items in BP, DEGs were related to potassium ion transport, the regulation of membrane potential, and the regulation of ion transmembrane transport. Enriched to 6 items in CC, which mainly included the voltage-gated potassium channel complex, potassium channel complex, cation channel complex, ion channel complex, and transporter complex, and so on. Enriching 13 items in MF, it mainly contained potassium channel activity, potassium ion transmembrane transporter activity, voltage-gated channel activity, carbohydrate binding, channel activity, etc.

As for GSEA, whole gene expressions were all included in analysis, the results was shown in **Figures 2B, C**; **Table 2**, which suggested that the cell cycle, DNA replication, graft versus host disease, nucleotide excision repair, and so on were enriched in control samples, while complement and coagulation cascades, Fc γ R–mediated phagocytosis, neuroactive ligand–receptor interaction, the NOD-like receptor (NLR) signaling pathway, gap junctions, etc., were enriched in IVDD samples.

**Table 2:** Gene set enrichment analysis results.

Description	setsize	Enrichment Score	NES	pvalue	p.adjust	qvalues
KEGG_CELL_CYCLE	124	-0.500739551	-2.27892	1.96E-09	1.80E-07	1.29E-07
KEGG_DNA_REPLICATION	36	-0.699169129	-2.49989	1.56E-08	9.59E-07	6.86E-07
KEGG_GRAFT_VERSUS_HOST_DISEASE	39	-0.638626883	-2.33148	9.25E-07	3.24E-05	2.31E-05
KEGG_NUCLEOTIDE_EXCISION_REPAIR	42	-0.618132469	-2.286	1.06E-06	3.24E-05	2.31E-05
KEGG_RIBOSOME	84	-0.615125806	-2.64052	1.00E-10	1.84E-08	1.32E-08
KEGG_SPLICEOSOME	125	-0.47405213	-2.16221	3.20E-08	1.47E-06	1.05E-06
KEGG_COMPLEMENT_AND_COAGULATION_CASCADES	69	0.499165382	1.991195	3.74E-05	0.000639	0.000457
KEGG_FC_GAMMA_R_MEDIATED_ PHAGOCYTOSIS	91	0.373040845	1.548242	0.003004	0.017823	0.012745
KEGG_NEUROACTIVE_LIGAND_RECEPTOR_ INTERACTION	268	0.342876817	1.647836	4.17E-05	0.000639	0.000457
KEGG_NOD_LIKE_RECEPTOR_SIGNALING_ PATHWAY	61	0.430642513	1.682186	0.002507	0.017084	0.012217
KEGG_OLFACTORY_TRANSDUCTION	367	0.316604209	1.575069	2.73E-05	0.000558	0.000399
KEGG_VASCULAR_SMOOTH_MUSCLE_ CONTRACTION	110	0.413013965	1.762641	0.000193	0.002088	0.001493

### 3.3 Identification and validation of characteristic genes

**Figure 3:**
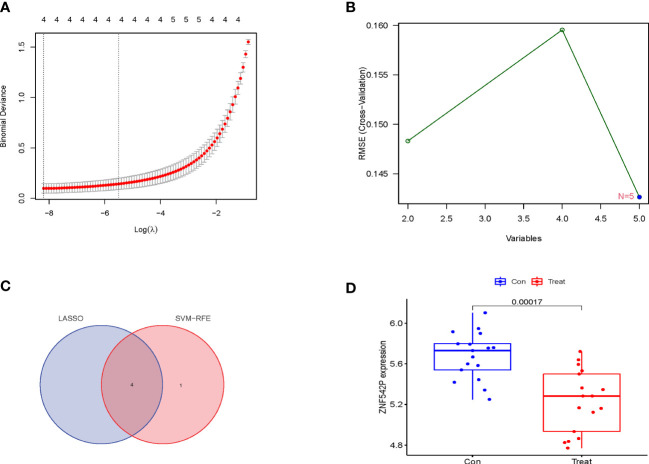
Identification and validation of characteristic genes for IVDD. **(A)** The characteristic genes were obtained *via* the LASSO logistic regression. **(B)** The characteristic genes were identified by the SVM-RFE algorithm. **(C)** Venn diagram showed the overlapping genes acquired by the LASSO logistic regression and the SVM-RFE algorithm. **(D)** the expression of ZNF542P in the validation set.

The LASSO logistic regression and SVM-RFE algorithms were used to identify characteristic genes from the 5 DEGs. Firstly, 4 characteristic genes, namely, ZNF542P, KCNH4, ASMTL-AS1, and TBC1D31, were obtained *via* the LASSO logistic regression (**Figure 3A**). Moreover, 5 genes, namely, ASMTL-AS1, LGALSL, ZNF542P, TBC1D31 and KCNH4, were identified by the SVM-RFE algorithm (**Figure 3B**). Finally, ZNF542P, KCNH4, ASMTL-AS1, and TBC1D31 were screened as characteristic genes through overlapping the genes acquired by the two algorithms (**Figure 3C**). In order to further test the expression levels of these 4 characteristic genes, we performed the differential expression analysis in the validation set and found that the expression of ZNF542P was downregulated (p = 0.00017) in IVDD samples compared to control samples (**Figure 3D**). The expression of KCNH4, ASMTL-AS1, and TBC1D31 was not statistically significant (p = 0.92, 0.23, 0.33). Thus, ZNF542P was considered as a key characteristic gene.

### 3.4 Estimation of diagnostic value of key characteristic gene

**Figure 4:**
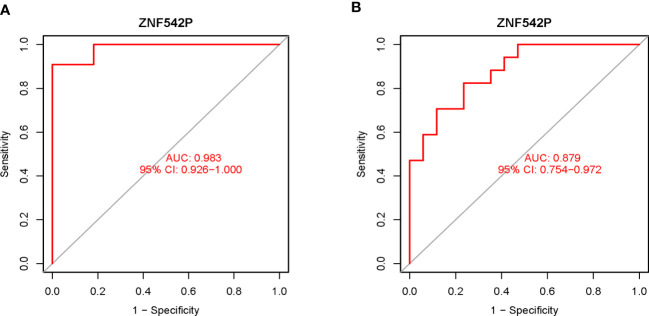
Estimation of the diagnostic value of the key characteristic gene. **(A)** The ROC curve analysis of ZNF542P in the training set. **(B)** The ROC curve analysis of ZNF542P in the validation set.

Based on the key characteristic gene identified in this study, we performed ROC curve analysis to estimate the clinical diagnostic value. As shown in **Figure 4**, the results showed that the AUC of ZNF542P was 0.983 (95%CI: 0.926–1.000) in the training set (**Figure 4A**), while AUC was 0.879 (95%CI: 0.754–0.972) in the validation set (**Figure 4B**), respectively. Both of the AUCs were greater than 0.7, which indicated that ZNF542P could be used as the diagnostic biomarker.

### 3.5 Nucleus pulposus cell culture and staining identification

**Figure 5:**
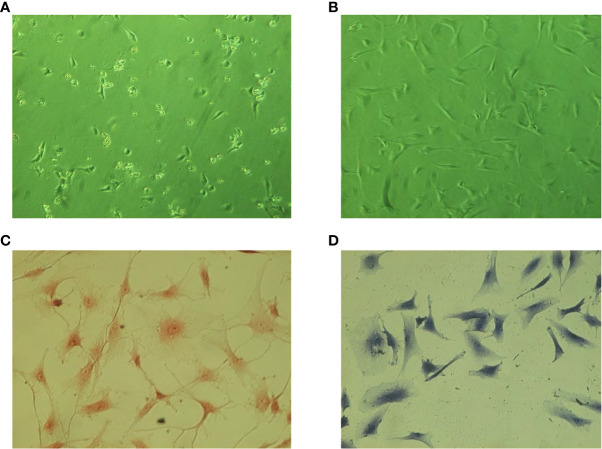
Cell culture and staining identification. **(A)** Cells 7 days after inoculation. **(B)** Cells 21 days after inoculation. **(C)** Safranine O staining of NP cells (×200). **(D)** Toluidine blue staining of NP cells (×200).

The primary NP cells began to adhere to the wall after 24 h (**Figure 5A**). The cells basically adhered to the wall 7 days later (**Figure 5B**). The cells were spindle shaped, with large and round nuclei and abundant cytoplasm. Compared with normal NP cells, the primary adherent time of degenerated NP cells was slightly longer. The cell morphology was poor and easy-to-appear aging cells after passage. Safranine O staining showed that the nucleus was red and cytoplasm staining was slight, indicating that cells could synthesize proteoglycans (**Figure 5C**). Toluidine blue staining showed that the nucleus was blue and cytoplasm staining was slight, demonstrating that cells could secrete glycosaminoglycans (**Figure 5D**).

### 3.6 The expression of the ZNF542P gene in nucleus pulposus cells

**Figure 6:**
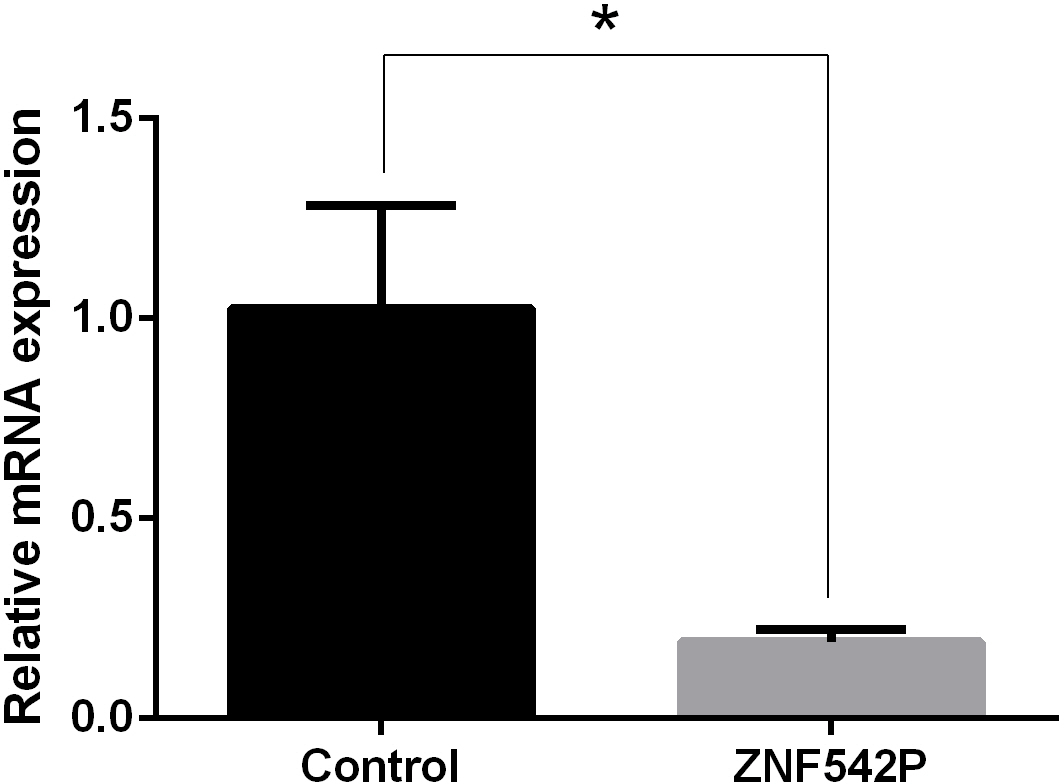
The expression of the ZNF542P gene. Data presented as mean ± SD, *P < 0.05, vs. control group; as evaluated by the t-test.

Compared with normal NP cells, the expression of the ZNF542P gene was decreased in degenerated NP cells (p < 0.05) shown in **Figure 6**.

### 3.7 The expression of NLRP3 and pro Caspase-1 in nucleus pulposus cells

**Figure 7:**
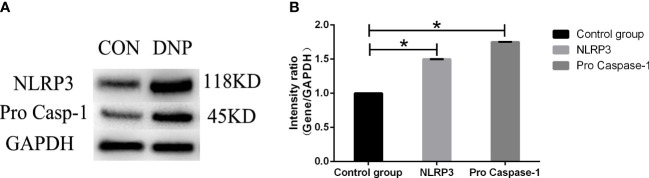
The expression of NLRP3 and pro Caspase-1. **(A)** The protein band. **(B)** The protein quantification. Data presented as mean ± SD, *P < 0.05, vs. control group, as evaluated by the *t*-test. CON, control;DNP, degenerated NP.

Compared with normal NP cells, the expression of NLRP3 and pro Caspase-1 were increased in degenerated NP cells shown in **Figure 7A, B**.

### 3.8 Correlation between key characteristic genes and immune cell infiltration

**Figure 8:**
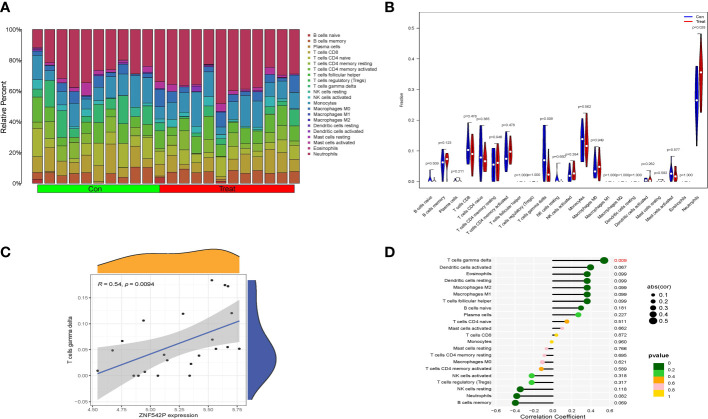
Correlation between key characteristic gene and immune cell infiltration. **(A)** Relative percentage of 22 immune cells in each samples. **(B)** Violin plot of the difference in each type of immune cell between IVDD and control samples. **(C, D)** The correlation analysis between key characteristic gene and various infiltrating immune cells.

The training set, including 11 IVDD samples and 11 control samples, was selected for the analysis of immune cell infiltration using the CIBERSORT algorithm, which was applied to calculate the relative percentage of 22 types of immune cells in each sample (**Figure 8A**). Compared with control samples, the results suggested that T cells gamma delta (γδT cells) were abnormally downregulated while neutrophils were upregulated in IVDD samples (**Figure 8B**). Moreover, the correlation analysis between the key characteristic gene and various infiltrating immune cells showed that the expression of ZNF542P was positively related to the proportions of γδT cells (R = 0.54, P = 0.0094), as shown in **Figures 8C, D**.

## 4 Discussion

IVDD is a common spine disease associated with many risk factors, such as genetic susceptibility (7), mechanical stress (8), trauma (9), heavy workload (10), smoking (11), obesity (12), and aging (13) that may result in the occurrence of IVDD. Previous studies have suggested that a lot of pathological processes including ECM degradation (14), systemic inflammation (15), oxidative stress (16), mitochondrial dysfunction (17), telomere shortening (18) and DNA damage (19), nutritional deprivation (20), abnormal mechanical load (21), and epigenetic changes (22) may lead to IVDD. Currently, the early diagnosis of IVDD is restricted. To identify key characteristic gene for IVDD and explore the correlations of key characteristic gene and immune cell infiltration, we acquired 3 IVDD-related gene expression profiles from the GEO database and screened 5 DEGs including 3 upregulated genes (KCNH4, LGALSL, and ASMTL-AS1) and 2 downregulated genes (ZNF542P and TBC1D31).

Subsequently, GO enrichment analysis was conducted on the DEGs. As a result, these DEGs were enriched in BP, CC, and MF. BP mainly included potassium ion transport, the regulation of membrane potential, and the regulation of ion transmembrane transport. CC mainly contained the voltage-gated potassium channel complex, potassium channel complex, cation channel complex, ion channel complex, and transporter complex. Simultaneously, MF mainly included potassium channel activity, potassium ion transmembrane transporter activity, voltage-gated channel activity, carbohydrate binding, and channel activity.

In addition, we found that 42 pathways were related to whole gene expressions. As revealed by the GSEA, those genes were mostly associated with complement and coagulation cascades, Fc γ R–mediated phagocytosis, neuroactive ligand–receptor interaction, the NOD-like receptor signaling pathway, gap junctions, and so on. A study showed that there are a total of 1,785 proteins in normal lumbar disks (NDs) and 1,775 proteins in degenerate disks (DDs). The ND primarily participated in ECM maintenance and basic metabolic pathways, whereas the unique proteins of the DD group were involved in inflammatory pathways (complement and coagulation cascades)and infective channels that support the recent theories of inflammaging and subclinical infection as pathogens of IVDD (23). Fc γ R IIIa (FcγR3A)–mediated phagocytosis, the regulation of Toll-like receptors (TLRs) by endogenous ligand, neutrophil, and platelet degranulation, was a significantly changed pathway in patients with modic changes (24). The neuroactive ligand–receptor interaction pathway was a significant pathway response to osmotic stimuli in the intervertebral disk (25). The NOD-like receptor (NLR) signaling pathway is closely related to immune response. A study indicated that thioredoxin-interacting protein (TXNIP) could aggravate IVDD through the NLRP3/Caspase-1/IL-1β signaling pathway (26). The activation of the TLR4/NLRP3 signaling axis could cause apoptosis, oxidative stress, and inflammatory responses in NP cells. It has been reported that the activation of the NF-κB/NLRP3 signaling pathway could decrease cellular functions of NP cells and promoted cell pyroptosis (27). NF-κB signaling activation improved NLRP3 inflammasome activity, and NLRP3 activation increased NF-κB signaling activation induced by TNF-α. NLRP3 inhibition or NLRP3 knockdown induced autophagic flux in the presence of TNF-α (28). In our study, the results of Western blot suggested that the NOD-like receptor signaling pathway was activated. Another study illustrated that high mechanical loads could affect the intercellular communication *via* gap junctions in healthy annulus fibrosus tissue, while gap junctions additionally control chondrocyte physiology (29).

Next, the overlapping genes, ZNF542P, KCNH4, ASMTL-AS1, and TBC1D31, were identified as characteristic genes *via* LASSO logistic regression and SVM-RFE algorithms. Moreover, we tested the expression levels of these 4 characteristic genes, and found that the expression of ZNF542P was downregulated in IVDD samples compared to control samples in the validation set, whereas the expression of KCNH4, ASMTL-AS1, and TBC1D31, there were no changes. Thus, ZNF542P was considered as a key characteristic gene and had a good clinical diagnostic value *via* ROC curve analysis.

So far, there are few studies on the function of ZNF542P. It may be involved in transcriptional regulation. As a marker of statin response *in vitro*, its change is mostly correlated with statin-induced alternation in cellular cholesterol ester. Intracellular cholesterol ester levels upon simvastatin treatment were raised after ZNF542P knocking-down in a human hepatoma cell line (30). In our study, the results of qRT-PCR showed that compared with normal NP cells, the expression of ZNF542P gene was decreased in degenerated NP cells.

In view of the importance of immune response in IVDD, we assessed the proportion and composition of infiltrating immune cells in IVDD samples and control samples and found that neutrophils and γδT cells were significantly different. In particular, neutrophils were significantly increased while γδT cells was significantly decreased in IVDD samples compared to control samples. Neutrophils, a type of polymorphonuclear leukocyte existing in the human immune system, are well known as a major participant during acute inflammation. However, recent evidence has shown that they also participate in chronic inflammatory and adaptive immune responses (31). An epidural inflammation ranging from acute invasion of neutrophils to the formation of chronic granulation tissue existed in most dogs with IVDD (32).

γδT cells, as a part of the innate immune system, are a subset of T cells that play a vital role in various diseases, such as COVID-19 virus infections (33), cancer (34), hematologic malignancies (35), autoimmune diseases (36), and central nervous system diseases (37). γδT cells could respond to many molecular signals and have the ability to induce multiple cytokines, for example, GM-CSF, IL-4, IL-17, IL-21, IL-22, and IFN-γ (38). Recent data suggested the γδT cells were also involved in IVDD. Immune infiltration analysis showed that the imbalance of neutrophils and γδT cells were significantly related to IVDD (39). Finally, we found that the expression of ZNF542P was positively correlated with the proportions of γδT cells.

## 5 Conclusion

In conclusion, 5 DEGs were screened between IVDD samples and control samples. Moreover, ZNF542P was identified as a key characteristic gene in IVDD based on bioinformatics and machine learning algorithms, which were both downregulated and tested in the validation set. Furthermore, we found that the expression of ZNF542P may be associated with the NOD-like receptor signaling pathway and the infiltration of γδT cells. Therefore, our study may contribute to the understanding of IVDD and may help in improving the early diagnosis of IVDD. However, further studies are needed to study the roles of ZNF542P in IVDD.

## Data availability statement

The original contributions presented in the study are included in the article/supplementary materials. Further inquiries can be directed to the corresponding authors.

## Ethics statement

The studies involving human participants were reviewed and approved by Tianjin Hospital Ethics Committee. The patients/participants provided their written informed consent to participate in this study.

## Author contributions

All authors listed have made a substantial, direct, and intellectual contribution to the work and approved it for publication.
